# Not always what closes best opens better: mesoporous nanoparticles capped with organic gates

**DOI:** 10.1080/14686996.2019.1627173

**Published:** 2019-06-26

**Authors:** Elena Añón, Ana M. Costero, Pablo Gaviña, Margarita Parra, Jamal El Haskouri, Pedro Amorós, Ramón Martínez-Máñez, Félix Sancenón

**Affiliations:** a Instituto Interuniversitario de Investigación de Reconocimiento Molecular y Desarrollo Tecnológico (IDM), Universitad Politècnica de València, Universitat de València, Valencia, Spain; b CIBER de Bioingeniería, Biomateriales y Nanomedicina (CIBER-BBN), Spain; c Instituto de Ciencia de Materiales (ICMUV), Universitat de València, Valencia, Spain; d Departamento de Química, Universitat Politècnica de València, Valencia, Spain

**Keywords:** Mesoporous nanoparticles, gated nanodevices, esterase controlled release, 10 Engineering and Structural materials, 102 Porous / Nanoporous / Nanostructured materials

## Abstract

Four types of calcined MCM-41 silica nanoparticles, loaded with dyes and capped with different gating ensembles are prepared and characterized. **N1** and **N2** nanoparticles are loaded with rhodamine 6G and capped with bulky poly(ethylene glycol) derivatives bearing ester groups (**1** and **2**). **N3-N4** nanoparticles are loaded with sulforhodamine B and capped with self-immolative derivatives bearing ester moieties. In the absence of esterase enzyme negligible cargo release from **N1, N3** and **N4** nanoparticles is observed whereas a remarkable release for **N2** is obtained most likely due to the formation of an irregular coating on the outer surface of the nanoparticles. In contrast, a marked delivery is found in **N1, N3**, and **N4** in the presence of esterase enzyme. The delivery rate is related to the hydrophilic/hydrophobic character of the coating shell. The use of hydrophilic poly(ethylene glycol) derivatives as gating ensembles on **N1** and **N2** enables an easy access of esterase to the ester moieties with subsequent fast cargo release. On the other hand, the presence of a hydrophobic monolayer on **N3** and **N4** partially hinders esterase enzyme access to the ester groups and the rate of cargo release was decreased.

## GRAPHICAL ABSTRACT

**Figure:**
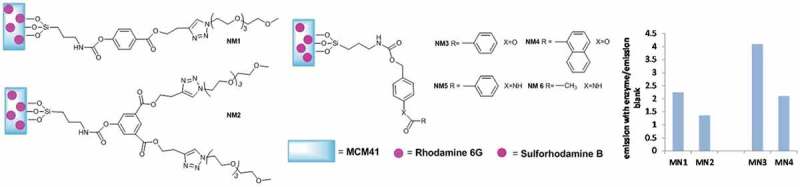

## 1. Introduction

The synthesis of gated nanodevices able to perform smart tasks has boosted in the last years and a number of examples have been published so far [1–4]. These gated nanodevices are normally composed by two subunits, namely a porous support, and certain (bio)molecules or supramolecular ensembles attached onto the external surface of the solid scaffold [5]. For the preparation of these gated nanodevices, usually the pores of the support are first loaded with selected cargos (i.e dyes, fluorophores, drugs, enzymes, small peptides) and then, the external surface is functionalized with the gated ensemble whose role is to inhibit delivery of the entrapped payload. Such nanodevices ideally show ‘zero’ release, yet the cargo is released upon application of an external stimuli [6–8].

As porous supports traditionally inorganic materials have been used even though other types of structures based in organic molecules (metal-organic frameworks (MOFs) and covalent organic frameworks (COFs)) are lately receiving great attention [9,10]. Even though different inorganic supports can be used [11], mesoporous silica of the MCM-41 family in the form of either micro- or nanoparticles, has been widely used due to its favourable features such as high load capacity, the presence of pores (ca. 2–3 nm), chemical stability, high specific surface area and volume and a relatively well-known functionalization chemistry using trialkoxysilane derivatives [12–17]. On the other hand, gating ensembles which responds to physical or chemical stimuli have been described. In this respect light, ultrasounds, magnetic fields, temperature, pH, changes in redox characteristics, cations, anions, small molecules, enzymes, and DNA fragments among others have been used as external stimuli to trigger cargo release from gated nanodevices [18]. These smart materials have been extensively used in controlled release protocols in (bio)medical applications, as new sensing platforms and in abiotic communication process mimicking those active in nature [19–23].

In spite of the amount of work devoted to the synthesis of smart-gated nanodevices and its potential applications in several fields there is a clear lack of systematic studies related to the chemical nature of the capping ensemble (size, polarity, hydrophobicity, hydrophilicity, possibility of intermolecular/intramolecular interactions, etc.) and how these features influence capping and the rate and efficiency of cargo release [24]. Such studies can be important as the required rate of delivery can be different depending on the application. For instance, whereas for sensing applications a quick cargo release is recommendable, in (bio)medical applications a more sustainable delivery can be necessary [25–29].

**Scheme 1.**
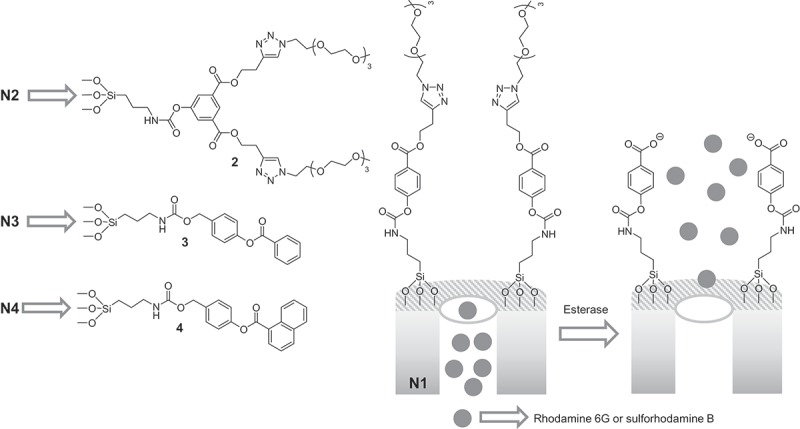
Structure of **N1** nanoparticles and the esterase-triggered release of the entrapped fluorophore. Also, the chemical structures of the other molecular gates used for the preparation of **N2-N4** nanoparticles are shown.

In this sense, we have previously reported the use of enzymes (esterases and amidases) as external stimuli with specific purposes [30,31] and during the development of these research we have observed that the characteristics of the organic molecules used as molecular gates have strong influence not only in the capping process but also in the release one. For all these reasons and due to our interest in the preparation of gated nanodevices [32–36] we decided to carry out a systematic study using ester with different characteristic to evaluate the influence of different factors in both capping and liberation steps. Thus, we report herein the synthesis of four different-gated systems (**N1-N4**) which are composed by mesoporous MCM-41 nanoparticles loaded with two different fluorophores (rhodamine 6G and sulforhodamine B) and capped with a series of four capping units containing ester groups (see Scheme 1). Two of the ester-capping units contained one (compound **1**) or two (derivative **2**) poly(ethylene glycol) chains whereas the others are formed by self-immolative structures containing aromatic rings (**3** and **4**).

## 2. Experimental section

### 2.1. General procedures

We used commercially available reagents without purification. Silica gel 60 F254 (Merck) plates were used for thin layer chromatography. Milli-Q ultrapure water was used for the sensing experiments. ^1^H and ^13^C nuclear magnetic resonance (NMR) spectra were recorded on a Bruker 300 MHz spectrometer (Bruker Corporation, Germany). Chemical shifts are reported in ppm with tetramethylsilane as an internal standard. High-resolution mass spectra were recorded in the positive ion mode on a TRIPLETOFT5600 (ABSciex, Canada). UV-vis absorption spectra were recorded on a Shimadzu UV-2101PC spectrophotometer at 293 K (Shimadzu Corporation, Japan). Fluorescence studies were carried out with a Varian Cary Eclipse (Agilent, United States) fluorescent spectrophotometer. Transmission electron microscopy (TEM) images were recorded with a JEOL-1010 microscope (Jeol Ltd., Japan) operated at 100 kV.

### 2.2. Synthesis of 13-azido-2,5,8,11-tetraoxatridecane (1b)

Tetraethyleneglycolmonomethylether (**1a**, 5 g, 24 mmol), trimethylamine (10.45 mL, 74 mmol) and 37.5 mL of dry dichloromethane (DCM) were placed in a round bottom flask and stirred at 0°C (ice/water bath) for 10 min, under argon atmosphere. Then a solution of *p*-toluenesulfonyl chloride (9.13 g, 48 mmol) in 71.9 mL of dry DCM was added dropwise and the reaction was stirred overnight (12 to 14 h) at room temperature. The reaction was extracted with water (3 × 25 mL). The organic phase was washed with brine (25 mL) and dried with anhydrous MgSO_4_. Evaporation of the solvent and purification using column chromatography (hexane:AcOEt 2:1 v/v) yield the tosylate (7.57 g, 87%) as orange oil. The tosylate was added to a solution of sodium azide (6.78 g, 0.1 mol) in 110 mL of dry dimethylformamide, and the mixture was stirred at room temperature overnight, under argon atmosphere. Water (100 mL) was added and the mixture was extracted with AcOEt (3 × 50 mL). The combined organic layers were washed with 10% NaHCO_3_ bicarbonate and brine. The organic phase was dried over anhydrous MgSO_4_ and evaporate to yield **1b** (2.14 g, 46%) as yellow oil. ^1^H NMR (300 MHz, chloroform-d): δ 3.67–3.59 (m, 12H), 3.55–3.50 (m, 2H), 3.37 (m, 2H,), 3.35 (s, 3H). ^13^C NMR (75 MHz, chloroform-d): δ 72.1,70.9,70.8,70.7,70.2,59.1,50.9. High resolution electrospray ionization mass spectrometry (HRMS (ESI)): m/z calculated for C_9_H_19_N_3_O_4_ (M^+^+1) 234.1448, found 234.1444.

### 2.3. Synthesis of but-3-yn-1-yl 4-hydroxybenzoate (1e)

4-hydroxybenzoic acid (**1c**, 0.67 g, 5 mmol) and **1d** (1.51 mL, 20 mmol) were dissolved in 5 mL of tetrahydrofuran (THF) in a round bottom flask. 0.1 mL of H_2_SO_4_ was added and the mixture was stirred at reflux during 24 h. The reaction was cooled at room temperature and poured over ice (50 mL). The solution was extracted with Et_2_O (3 × 25 mL) and the combined organic layers were washed with 10% NaHCO_3_ (2 × 25 mL) and brine (25 mL) and dried over anhydrous MgSO_4_. The solvent was evaporated and the product purified using column chromatography (hexane: AcOEt 1:2 v/v) to yield **1e** (0.45 g, 47%) as a brown solid. ^1^H NMR (300 MHz, chloroform-d): δ 7.95 (d, *J*= 8.9 Hz, 2H), 6.87 (d, *J*= 8.9 Hz, 2H), 4.40 (t, *J*= 6.8 Hz, 2H), 2.65 (td, J = 6.8, 2.7 Hz, 2H), 2.03 (t, *J*= 2.7 Hz, 1H). ^13^C NMR (75 MHz, chloroform-d): δ 167.0,161.0,132.5,122.3,115.7,80.5,70.4,62.9,20.2,19.5 ppm. High resolution electrospray ionization mass spectrometry (HRMS (ESI)): m/z calculated for C_11_H_10_O_3_ (M^+^+1) 191.0703, found 191.0701.

### 2.4. Synthesis of di(but-3-yn-1-yl) 5-hydroxyisophthalate (2b)

From 5-hydroxyisophthalic acid (0.91 g, 5 mmol) and **1d** (1.51 mL, 20 mmol) following the procedure described in the synthesis of **1e**, compound **2b** was obtained as yellow solid (0.61 g, 43%). ^1^H NMR (300 MHz, chloroform-d): δ 8.28 (t, *J*= 1.5 Hz, 1H), 7.77 (d, *J*= 1.5 Hz, 2H), 6.06 (s, 1H,), 4.45 (t, *J*= 6.8 Hz, 4H,), 2.68 (dt, *J*= 6.8, 2.7 Hz, 4H), 2.04 (t, *J*= 2.7 Hz, 2H). ^13^C NMR (75 MHz, chloroform-d): δ 165.8,156.6,132.1,123.4,121.4,70.6,63.4,31.4,19.5. High resolution electrospray ionization mass spectrometry (HRMS (ESI)): m/z calculated for C_16_H_14_O_5_ (M^+^+1) 287.0914, found 287.0915.

### 2.5. Synthesis of compound 1

Seven milligrams of copper acetate and 14 mg of sodium ascorbate in 0.58 mL of water were added to a solution of compound **1b** (230 mg, 1 mmol) and compound **1e** (210 mg, 1.1 mmol) in a mixture of THF (2.48 mL) and water (1.42 mL). The reaction was stirred during 24 h at room temperature. Water was added (10 mL) and the reaction extracted with DCM (3 × 5 mL). The organic layers were washed with water and brine (2 × 5 mL) and dried over anhydrous MgSO_4_. The solvent was evaporated and purification using column chromatography (hexane:EtAcO 3:7 v/v) gave **1** (310 mg, 72%) as a yellow oil. ^1^H NMR (300 MHz, chloroform-*d*): δ 7.85 (d, *J*= 7.8 Hz, 2H), 7.61 (s, 1H), 6.87 (d, *J*= 7.8 Hz, 2H), 4.54–4.45 (m, 4H), 3.79 (dd, *J*= 5.5, 4.4 Hz, 2H), 3.62–3.47 (m, 12H), 3.32 (s, 3H), 3.16 (t, *J*= 6.4 Hz, 2H). ^13^C NMR (75 MHz, chloroform-*d*): δ 166.8,162.4,144.4,132.1,123.5,121.3,115.9,72.1,70.8,70.7,70.6,70.5,69.7,63.6,59.2,50.2,25.8. High resolution electrospray ionization mass spectrometry (HRMS (ESI)): m/z calculated for C_20_H_29_N_3_O_7_ (M^+^+1) 424.2078, found 424.2080.

### 2.6. Synthesis of compound 2

From 20 mg of copper acetate and 40 mg of sodium ascorbate in 1.66 mL of water and a solution of compound **1b** (200 mg, 0.86 mmol) and compound **2b** (100 mg, 0.34 mmol) in a mixture of THF (3.34 mL) and water (1.67 mL) following the procedure previously described, compound **2** (0, 17 g, 65%) was isolated as yellow oil. ^1^H NMR (300 MHz, chloroform-*d*): δ 8.05 (bs, 1H), 7.61 (s, 2H), 7.59 (d, *J*= 1.5 Hz, 2H), 4.54 (t, *J*= 5.0 Hz, 4H), 4.48 (t, *J*= 5.0 Hz, 4H), 3.80 (t, *J*= 5.0 Hz, 4H), 3.62–3.45 (m, 24H), 3.27 (s, 6H), 3.14 (t, *J*= 5.0 Hz, 4H). ^13^C NMR (75 MHz, chloroform-*d*): δ 165.8,157.8,144.1,131.9,123.4,122.3,121.3,72.2,71.0,70.9,70.8,70.6,69.8,64.4,59.2,50.6,25.8 ppm. High resolution electrospray ionization mass spectrometry (HRMS (ESI)): m/z calculated for C_34_H_52_N_6_O_13_ (M^+^+1), 753.3671, found 753.3622.

### 2.7. Synthesis of 4-(hydroxymethyl)phenyl benzoate (3a)

4-hydroxybenzylalcohol (0.25 g, 2 mmol) was added to a solution of KOH (0.11 g, 2 mmol) in ethanol (5 mL). The mixture was stirring during 1 h at room temperature, and then benzoyl chloride (0.23 mL, 2 mmol) was added. The reaction was stirred overnight. The solvent was evaporated and re-solved in EtAcO (15 mL). The organic layer was washed with water (2 × 5 mL) and brine (2 × 5 mL) dried over anhydrous MgSO_4_, the solvent was evaporated and the solid was purified using column chromatography (hexane:EtAcO 2:1 v/v) to yield **3a** (0.17 g, 38%) as white solid. ^1^H NMR (300 MHz, chloroform-d): δ (ppm) 8.21 (d, *J*= 9.0 Hz, 2H), 7.65 (t, *J*= 9.0 Hz, 1H), 7.52 (t, *J*= 9.0 Hz, 2H), 7.44 (d, *J*= 9.0 Hz, 2H), 7.22 (d, *J*= 9.0 Hz, 2H), 4.73 (s, 2H). ^13^C NMR (75 MHz, chloroform-d): δ 165.7,150.8,139.0,134.0,130.6,129.9,129.0,128.5,122.2,65.2ppm.

### 2.8. Synthesis of 4-(hydroxymethyl)phenyl 1-naphthoate (4a)

4-hydroxybenzylalcohol (0.41 g, 3.31 mmol) was added to a solution of KOH (0.19 g, 3.31 mmol) in ethanol (17 mL) and stirred during 1 h, at room temperature. Then, naphthaloyl chloride (in 2 mL of ethanol), previously formed (1-naphthoic acid (0.57 g, 3.31 mmol) and thionyl chloride (4.8 mL, 66.2 mmol) were added to a round bottom flash and the mixture was warmed under reflux during 210 min, then, the solvent was evaporated and the residue was washed with ethyl ether, filtered and the organic layer was concentrated to yield the chloride acid) was added. The reaction was stirred under reflux during 3 h. The solvent was evaporated and re-solved in EtAcO (20 mL). The organic layer was washed with water (2 × 10 mL) and brine (2 × 10 mL), dried over anhydrous MgSO_4_, evaporated and purified using column chromatography (hexane/ethyl acetate from 2:1 to 1:1 v/v) to yield **4a** (0.33 g, 36%) as yellow solid. ^1^H NMR (300 MHz, dimethylsulfoxide-d6): δ 8.83 (d, *J*= 8.5 Hz, 1H), 8.45 (dd, *J*= 7.3, 1.3 Hz, 1H), 8.33–8.28 (m, 1H), 8.10 (dd, *J*= 7.9, 1.8 Hz, 1H), 7.76–7.62 (m, 3H), 7.45 (d, *J*= 8.5 Hz, 2H), 7.33 (d, *J*= 8.5 Hz, 2H), 5.28 (t, *J*= 5.7 Hz, 1H), 4.56 (d, *J*= 5.7 Hz, 2H). ^13^C NMR (75 MHz, dimethylsulfoxide-d6): δ 165.8,149.7,140.7,134.6,133.8,131.3,130.0,129.3,128.6, 128.0,126.9,125.9,125.4,122.0,62.8 ppm. High resolution electrospray ionization mass spectrometry (HRMS (ESI)): m/z calculated for C_18_H_14_O_3_ (M^+^+1) 279.1016, found 279.1015.

### 2.9. Synthesis of carbamates 3 and 4

In a round bottom flask, compounds **3a** or **4a** (1 equiv.) was dissolved in 15 mL of THF and 60% sodium hydride (2 equiv.) was added under argon atmosphere. The mixture was stirred for 30 min and then 3-(triethoxysilyl)propyl isocyanate (**3b**, 1 equiv.) was added. The mixtures were refluxed during the optimized time. The reaction was cooled at room temperature and then centrifuged at 11,000 rpm during 3 min. Yields: 66 and 50 for **3** and **4**, respectively. The supernatant was concentrated and used directly for functionalization of the external surface of the loaded support (see below the synthesis of **N3-N4** nanoparticles).

### 2.10. Synthesis of mesoporous MCM-41 nanoparticles

Cetyltrimethylammonium bromide (CTABr, 1.00 g, 2.74 mmol) was dissolved in 480 mL of deionized water before adding 3.5 mL of a solution of NaOH 2M until achieving pH 8. Then, the solution was heated to 80°C and tetraethyl orthosilicate (TEOS) (5.00 mL, 2.57 × 10^−2^ mol) was added dropwise at maximum stirring. The mixture was stirred for 2 h at 80°C. A white precipitate was obtained and isolated by centrifugation. The solid was washed with deionized water and ethanol till obtaining neutral pH in the solution, and dried at 60°C (MCM-41 as-synthesised). To prepare the final porous material (MCM-41), the as-synthesised solid was calcined at 550°C using an oxidant atmosphere for 5 h.

### 2.11. Synthesis of N1 and N2

Calcined MCM-41 (100 mg) and rhodamine 6G (100 mg, 0.17 mmol) were suspended in acetonitrile (20 mL). Then, the mixture was stirred at reflux during 2 h using a Dean–Stark (to eliminate 10 mL of water) and then kept at room temperature overnight. Afterward, 3-(triethoxysilyl)propylisocianate (**3b**, 120 mg, 0.5 mmol) was added and the mixture was stirred during 5 h at room temperature. Then, sodium (in two small parts) and **1** or **2** (0.5 mmol) were added and the mixture was stirred at room temperature overnight. Finally, solids **N1** and **N2** were washed with deionized water and dried at 70°C overnight.

### 2.12. Synthesis of N3-N4

Calcined MCM-41 (100 mg) and sulforhodamine B (100 mg, 0.17 mmol) were suspended in acetonitrile (20 mL). Then, the mixture was stirred at reflux during 2 h using a Dean–Stark apparatus (to eliminate 10 mL of water) and then kept at room temperature overnight. Afterward, triakoxysilane derivatives **3**–**4** (0.5 mmol) were added and the mixture was stirred at room temperature overnight. Finally, solids **N3-N4** were washed with deionized water and dried at 70°C overnight.

## 3. Results and discussion

### 3.1. Design and synthesis of the capped nanoparticles

The four prepared capped nanoparticles (**N1-N4**) are shown in Scheme 1. **N1** and **N2** used capping ensembles which comprises poly(ethylene glycol) chains linked to a benzene ring through ester linkages that could be hydrolyzed using esterase enzyme [37–40]. Capping ensemble in **N1** is less bulky than that presented in **N2**, which contained two poly(ethylene glycol) chains. On the other hand, the capping units in **N3** and **N4** are designed to suffer a self-immolative process in the presence of esterase enzyme [41–43]. Besides, both ensembles contain aromatic rings (benzene in **N3** and naphthalene in **N4**) which could generate π-stacking interactions.

**Scheme 2.**
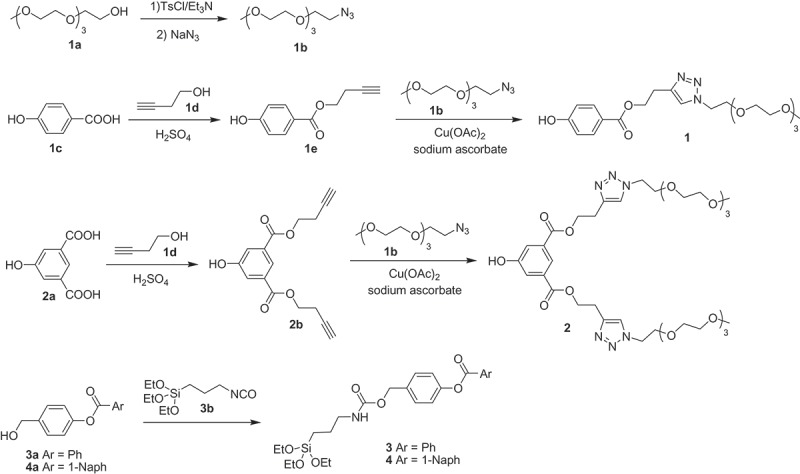
Synthetic routes used for the preparation of capping compounds **1**–**4.**

Poly(ethylene glycol) derivatives **1** and **2**, used for the preparation of **N1** and **N2** nanoparticles, were synthesized as shown in Scheme 2. Tetraethyleneglycol monomethyl ether (**1a**) was transformed into the corresponding azide derivative (**1b**). Then, reaction of 4-hydroxy benzoic acid (**1c**) or 5-hydroxyisophthalic acid (**2a**) with one or two equivalents of 3-butin-1-ol (**1d**) under Fischer esterification conditions gave the corresponding esters (**1e**) and (**2b**). Finally, Cu(I)-catalyzed alkyne-azide Huisgen cycloaddition between **1b** and **1e** yielded the final poly(ethylene glycol) derivative **1** [44–47]. The same cycloaddition carried out with **1b** and **2b** yielded the capping molecule **2**. Carbamate derivatives **3** and **4**, used for the preparation of **N3** and **N4** materials were prepared following synthetic routes shown in Scheme 2. Compounds **3a** and **4a** were obtained by reacting 4-hydroxy benzylic alcohol with benzoyl chloride and 1-naphthoyl chloride, respectively. Then, alkoxysilane derivatives **3** and **4** were obtained by the reaction between **3a** and **4a** with sodium hydride and (3-isocyanatopropyl)triethoxysilane (**3b**) in moderate yields (66% and 50% for **3** and **4,** respectively). To avoid tedious separation process and shorten the time for the final nanoparticle preparation, the crude reactions obtained in the synthesis of **3** and **4 **were not purified and used directly to functionalize the dye loaded mesoporous nanoparticles (see below). All the prepared products were characterized by ^1^H and ^13^C NMR and MS (see experimental section).

Mesoporous silica nanoparticles of the MCM-41 family were prepared following well-known procedures using TEOS as inorganic precursor and cetyltrimethylammonium bromide as structure directing agent [48]. The solid was calcined to eliminate the structure directing agent (**N0** nanoparticles). For the preparation of **N1** and **N2**, the pores of the inorganic support were loaded with rhodamine 6G by stirring a high concentrated solution of the dye in the presence of **N0**. Then, the external surface of the loaded support was functionalized with (3-isocyanatopropyl)triethoxysilane and poly(ethylene glycol) derivatives **1** and **2** were grafted onto the external surface by the reaction with the isocyantopropyl moieties through the formation of carbamate bonds. The pink solids obtained (**N1** and **N2**) were filtered, washed with water, and dried at 70°C for 12 h.

**N3** and **N4** nanoparticles were prepared following a similar procedure. In this case, the pores of the calcined **N0** nanoparticles were loaded with sulforhodamine B. Then, the pores were capped upon addition of the crude reaction obtained in the synthesis of trialkoxysilane derivatives **3** and **4** (containing a 66 and a 50% of **3** and **4** respectively). The final capped nanoparticles were filtered, exhaustively washed with water to remove any starting material and dried at 70°C for 12 h.

### 3.2. Characterization of the capped nanoparticles

**Figure 1.**
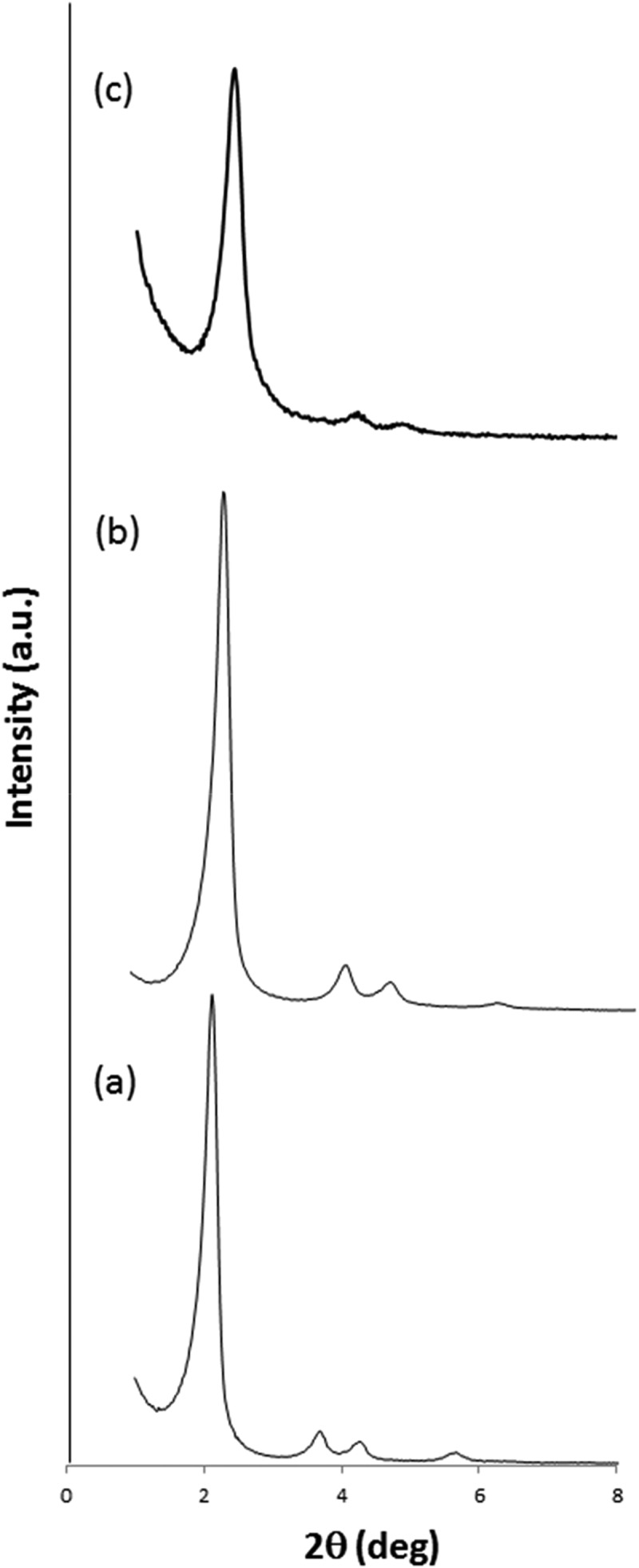
PXRD patterns of (a) the nanoparticulated MCM-41 as-synthesized, (b) calcined **N0** nanoparticles, and (c) **N3** nanoparticles.

The as synthesized MCM-41 scaffold, the calcined nanoparticles (**N0**) and final solids **N1-N4** were characterized following standard procedures such as powder X-ray diffraction (PXRD), TEM, elemental analysis and porosimetry. PXRD measurements, carried out on the as synthesized materials, showed clearly the presence of a mesoporous structure that persisted in the final solids regardless of the loading process with the dye, and further functionalization with capping molecules. Figure 1 shows the PXRD pattern of as synthesized mesoporous nanoparticles, **N0** and **N3** solids. PXRD of MCM-41 nanoparticles as-synthesised showed four low-angle peaks, typical of a hexagonal-ordered pore array that can be indexed as (10), (11), (20) and (21) Bragg reflections. A shift of the (10) peak (from *d* spacing values of 4.11 to 3.75 nm) and a remarkable broadening of the (11) and (20) in calcined MCM-41 (**N0**) were observed and ascribed to further condensation of silanols during the calcination step. The presence of the characteristic (10) reflection in the diffraction patterns obtained for solid **N3** indicated that the mesoporous structure was preserved throughout the filling process with sulforhodamine B and the anchoring of capping ensemble **3**. Similar features were observed in the PXRD patterns of **N1, N2,** and **N4** nanoparticles (data not shown).

**Figure 2.**
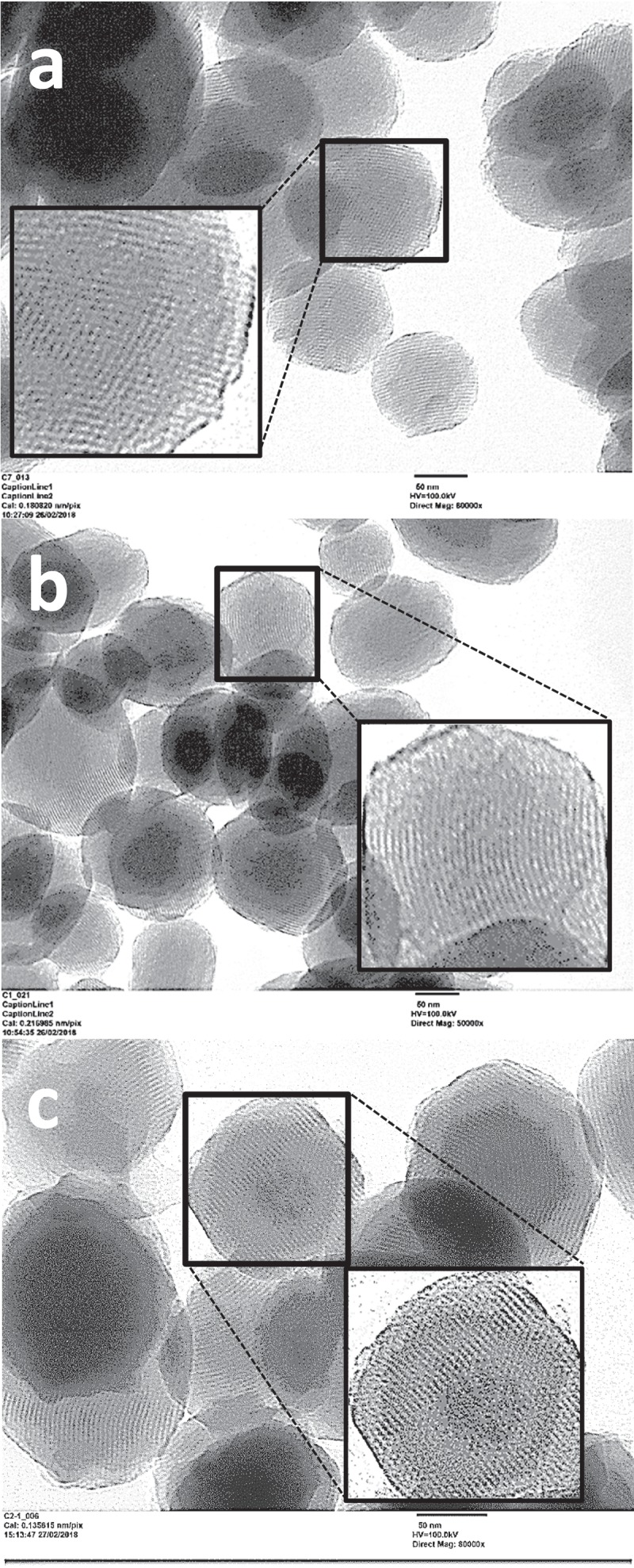
Representative TEM images of the functionalized nanoparticles: (a) **N1**, (b) **N3** and (c) **N4.**

TEM images of the final materials (see Figure 2 for **N1, N3**, and **N4** nanoparticles) confirmed the preservation of the highly ordered hexagonal mesostructure. All samples present a morphology defined by pseudo-spherical silica-based particles with diameters between 80 and 140 nm. The possible partial aggregation of these primary nanoparticles defines large interparticle textural type meso/macropores. The intraparticle order is clearly observed as a regular alternance of black and white spots or lines, that correspond to the mesopores viewed along the (10) plane or in the perpendicular direction.

The N_2_ adsorption–desorption isotherms of the calcined nanoparticles (**N0**) and of the loaded and gated solids (**N1-N4**) are shown in the Supporting Information. Besides, Table 1 shows selected parameters of the prepared nanoparticles. BET (Brunauer, Emmett, Teller) [49], BJH (Barret, Joyner, Halenda) [50] and NLDFT (non-linear density functional theory) [51] models have been applied. Although the BJH method is the standard procedure for calculating the pore size distribution in mesoporous materials, it has been shown that this model leads to underestimates. Then, we have also estimated the pore size by using the NLDFT model [52] for cylindrical mesopores. Although there are no important relative differences, it is accepted that the pore size values provided by the NLDFT model are more realistic. Calcined nanoparticles showed, as expected, a high BET surface area (1005.7 m^2^ g^−1^) and a remarkable pore volume (0.56 cm^3^ g^−1^) typical of mesoporous MCM-41-like solids. The BET surface area and BJH intraparticle mesopore volume decrease significantly for solids **N1** and **N2** when compared to that of the calcined support (**N0**). These decreases could be ascribed to an efficient pore loading and to an effective grafting of the capping molecules onto the external surface of the nanoparticles. On the other hand, the **N1** and **N2** intraparticle pore size (BJH and NLDFT) slightly decrease respect to the original in initial support (**N0**). These mesopores, still accessible to nitrogen, correspond to the fraction of pores in which the closing of the nano gates is not complete. However, note that the proportion of these pores is very low according to the small BJH volume. For **N3** and **N4** nanoparticles higher BET areas and BJH intraparticle pore volumes, when compared to those measured for **N1** and **N2**, were observed. These higher values could be ascribed to the functionalization of the external surface with the crude reaction which contained only 66% and 50% of alkoxysilane derivatives **3** and **4**. Taking into account these yields, nearly one-half of the grafted molecules were alkoxysilanes bearing small isocyanatopropyl moieties which were unable to induce proper pore blocking and, as a consequence, higher areas and pore volumes were obtained. Besides, the small size of the capping molecule, when compared to those used for the preparation of **N1** and **N2**, could also account for the obtained values. On the other hand, the intraparticle (textural) porosity seems to be less affected.

**Table 1.** *d* spacing values from XRD, BET specific surface values, pore volumes and pore sizes calculated from the N_2_ adsorption-desorption isotherms for calcined (**N0**) and **N1-N4** nanoparticles.

Solid	*d* spacing (nm)^[a]^	S_BET_ (m^2^ g^−1^)	Mesopore size (nm)^[b]^	Mesopore volume (cm^3^ g^−1^)^[c]^	Mesopore size (nm)^[c]^	Textural pore volume (cm^3^ g^−1^)^[c]^	Textural pore size (nm)^[c]^
N0	3.75	1005.7	3.42	0.56	2.30	0.30	46.9
N1	3.64	79.6	2.60	0.04	2.12	0.39	25.3
N2	3.61	54.8	2.99	0.02	2.21	0.18	22.1
N3	3.66	118.3	2.71	0.02	2.16	0.48	39.7
N4	3.64	535.6	3.34	0.30	2.22	0.42	55.8

[a] *d* spacing values from the (10) peak in the XRD patterns. [b] Intraparticle cylindrical mesopore size determined through NLDFT model. [c] Pore volumes and sizes estimated by the BJH model applied to the adsorption branch of the isotherm. The mesopore volume and size associated to the intraparticle surfactant-generated pores have been estimated from P/P_0_ values <0.8. The textural pore volume and size (due to the voids formed through the packing of the pseudo-spherical particles) have been determined from P/P_0_ values >0.8.

The amount of dye-loaded inside the pores and of capping ensemble in the final **N1-N4** nanoparticles, calculated using elemental analysis and UV-visible, are shown in Table 2. The amount of loaded dye was calculated by dissolution of the corresponding nanoparticles (previously suspended in water) using sodium hydroxide and then measuring the emission intensity of the resultant solution (for rhodamine 6G at 550 nm (λ_exc_ = 520 nm) whereas for sulforhodamine B at 585 nm (λ_exc_ = 565 nm)). Elemental analysis carried out with the six prepared nanoparticles indicated the total organic content. The difference between the total organic content (assessed from elemental analysis data) and the dye loaded (determined using fluorescence measurements) yielded the amount of capping ensemble in each of the final nanoparticles. Besides, for **N3-N4** nanoparticles, the capping ensemble content was corrected taking into account the yields of the reaction used for the preparation of compounds **3**–**4**. As could be seen in Table 2, the amount of dye loaded in **N2-N4** nanoparticles was quite similar (1–1.6% wt) whereas for **N1** lower content was assessed.

**Table 2.** Organic matter content for **N1-N4** nanoparticles.

Solid	Dye amount (% wt)	Capping ensemble (mg g^−1^)^[a]^	Yield of capping molecules synthesis	Capping ensemble (mmol g^−1^)^[b]^
N1	0.57	238	100	0.47
N2	0.95	552	100	0.85
N3	1.05	587	66	1.24
N4	1.17	575	50	0.79

[a] from elemental analysis; [b] values corrected taking into account the yield of the synthesis of capping molecules 3–6.

The existence of a possible π-π stacking in **N3** and **N4** was evaluated using UV measurements both in solid state and in solution at different concentration. The expected shift of the absorption bands, generally attributed to interactions between aromatic rings, was clearly observed not only in solution but also in solid phase [53] (see Supporting Information, figures S-11, S-12, and S-13)

### 3.3. Enzyme-triggered controlled release performances of the prepared nanoparticles

**Figure 3.**
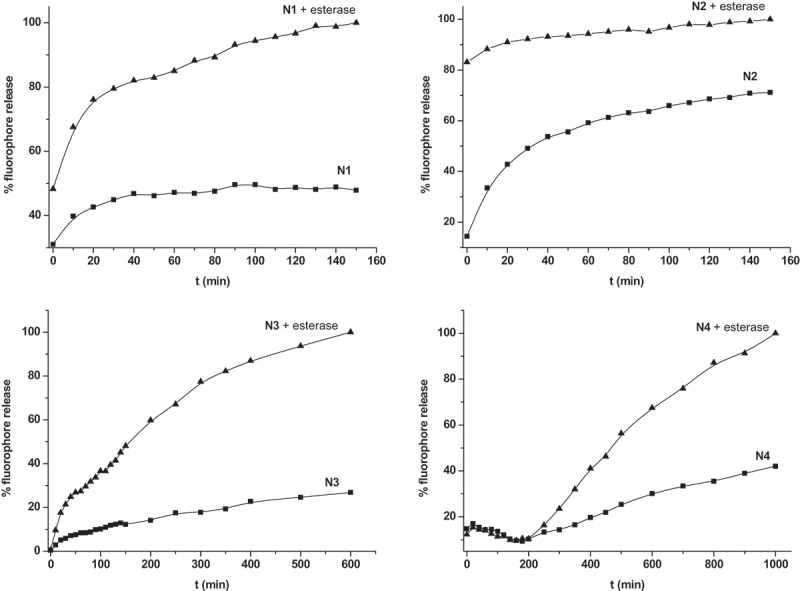
Release profiles of fluorophores (rhodamine 6G for **N1** and **N2** and sulforhodamine B for **N3** and **N4**) in the absence and in the presence of esterase (6 mg) in TRIS buffer (260 mM, 8.06 pH).

The controlled release of the **N1-N4** nanoparticles in the absence and in the presence of esterase enzyme was studied. In a typical experiment, 1 mg of the corresponding nanoparticles were suspended in 3 mL of TRIS (260 mM, pH 8.06) in the presence or absence of the esterase. Suspensions were stirred at room temperature for 24 h and aliquots of the samples were taken at a given time. The solid was then removed by centrifugation and dye delivery was monitored by measuring the emission of rhodamine 6G at 550 nm (λ_exc_ 520) or of sulforhodamine B at 585 nm (λ_exc_ 565). The dye delivery profiles obtained for **N1-N4** are depicted in Figure 3. In the absence of enzyme dye release from **N1** and **N3** nanoparticles was very small and remained constant over the measuring time. On the other hand, dye release from **N4** nanoparticles gradually increased with time (reaching ca. 40% after 1000 min). In contrast, the release of rhodamine 6G from **N2** nanoparticles in the absence of external trigger (esterase) was remarkable. This fact was tentatively ascribed to the presence of two bulky chains in *meta* positions in the phenyl ring that adopt a spatial arrangement not suitable for the formation of a monolayer. In fact, the molecular gate used in the preparation of **N2** shows an angular geometry clearly different than those of the other molecules that are more planar (see Supporting Information, Figure S-10). As a consequence, the external surface of the nanoparticles is coated with an irregular shell that is unable to effectively retain the dye in the inner of the porous system. By contrast, **N1, N2**, and **N3**, where the substituents in the phenyl ring are in *para* position, the spatial disposition (more planar) of the chains is more appropriate for the formation of uniform monolayer coatings. Comparing the release profiles in the absence of esterase for **N1** and **N2** nanoparticles and their gating ensemble contents (238 and 552 mg g^−1^ solid for **N1** and **N2** respectively) it is evident that a more efficient pore blocking is achieved with molecules able to produce uniform monolayer coatings, whereas the amount of grafted gate has minimal contribution in this occasion.

In the presence of esterase a marked payload release was observed for **N1-N4** (Figure 3). However, different release profiles were obtained which are related to the nature of the gating ensemble. The faster dye delivery was obtained with **N2** nanoparticles; after 10 min ca. 90% of the total delivery was observed. This fact could be ascribed to the non-uniform monolayer of gating ensemble formed in the outer surface of the nanoparticles, which could allow esterase enzyme to reach faster the ester moieties of the gating ensemble inducing a marked and instantaneous release. As a clear contrast, a more progressive rhodamine 6G release was observed for **N1** (ca. 65% of the total delivery after 10 min). This progressive delivery was ascribed to the presence of a more dense poly(ethylene glycol) hydrophilic coating around pore outlets that was hydrolyzed more slowly by esterase enzyme.

In contrast, esterase-triggered dye release from **N3** and **N4** nanoparticles were the slowest of all the prepared materials; 65% of the total delivery was reached after ca. 250 and 600 min for **N3** and **N4** respectively. This slower release rate is tentatively ascribed to the formation of a dense hydrophobic monolayer around pore outlets. In spite of the fact that gating molecules in **N3** and **N4** are small (when compared with the bulky poly(ethylene glycol) derivatives used in **N1** and **N2**) the hydrophobic character of the monolayer could difficult the access of the esterase enzyme to the ester bonds, which after hydrolysis triggered-on the self-immolative cascade inducing pore opening. Also, the possible presence of π-stacking interactions between naphthalene moieties located in the gating ensemble of **N4** nanoparticles could account for the slower release observed [53]. Besides, the presence of a marked external surface (as a consequence of the moderate yield obtained in the synthesis of **3** and **4**) in both nanoparticles (**N3** and **N4**) could allow certain esterase adsorption which defaulted, to some extent, cargo delivery.

## 4. Conclusions

Herein we present the synthesis and characterization of four types of calcined MCM-41 silica nanoparticles loaded with dyes (rhodamine 6G and sulforhodamine B) and capped with several gating ensembles of different sizes and shapes, containing hydrophilic and hydrophobic moieties and equipped with enzyme-sensible ester bonds. The formation of dense monolayers around pore outlets is a crucial factor in order to obtain a proper blocking and a negligible release. In this respect, the capping efficiency of the bulky and branched poly(ethylene glycol) derivative **2** is low and aqueous suspensions of **N2** nanoparticles presented a marked dye release in the absence of the external trigger (esterase enzyme). Other smaller gating molecules generated dense monolayer around the pore outlets in nanoparticles effectively inhibiting cargo release. The presence of esterase enzyme induced cargo release from **N1-N4** nanoparticles due to the hydrolysis of ester bonds located in the structure of the capping ensemble. However, the rate of the enzyme-triggered release is clearly related to the hydrophilic/hydrophobic character of the coating while the size of the sensing ensemble less important. In this respect, the hydrophilic coating in **N1** is more accessible to esterase, and this fact induced faster release when compared with the more hydrophobic capping ensembles in **N3** and **N4**, despite the sizes of the molecular gates used in the later nanoparticles are smaller than those used for **N1**. In addition, the relatively low amount of capping ensemble/g in the case of **N1**, when compared to **N2** and **N3**, also could contribute for a quicker release. In short, different parameters including the capping ensembles density at the surface, their sizes and also more fine chemical details as their hydrophobic/hydrophilic character or steric conformations must be taking into account in order to control in a sensitive way the release processes from mesoporous materials. We think that systematic studies, similar to that shown here, can help researchers working in this excitant multidisciplinary field. By selecting the nature of the capping ensemble it is possible to modulate the rate of cargo delivery, which is of importance for certain applications. Several additional efforts into this direction are currently carried out in our research group.
