# Supplementary material for: Not always what closes best opens better: mesoporous nanoparticles capped with organic gates
Source: Sci Technol Adv Mater. 2019 Jun 26;20(1):699–709. doi: 10.1080/14686996.2019.1627173 (PMC6598471; doi:10.1080/14686996.2019.1627173)

Not always what closes best opens better: Mesoporous nanoparticles capped with organic gates

Elena Añón,[1,3] Ana M. Costero,[1,3]* Pablo Gaviña,[1,3] Margarita Parra,[1,3] Salvador Gil, [1,3] Jamal El Haskouri,[4] Pedro Amorós,[4] Ramón Martínez-Máñez[2,3]* and Félix Sancenón[2,3]

[1]Instituto Interuniversitario de Investigación de Reconocimiento Molecular y Desarrollo Tecnológico (IDM). Universitad Politècnica de València, Universitat de València, Doctor Moliner 50, Burjassot, 46100, Valencia, Spain.

[2] Departamento de Química, Universitat Politècnica de València, Camí de Vera s/n, 46022, Valencia (Spain).

[3]CIBER de Bioingeniería, Biomateriales y Nanomedicina (CIBER-BBN) (Spain).

[4] Instituto de Ciencia de Materiales (ICMUV), Universitat de València, P.O. Box 2085, Paterna, 46071, Valencia, Spain.

E-mail: ana.costero@uv.es

Figure S1. ^{1}H NMR and ^{13}C NMR spectrum of compound **1** in $CDCl_3$ at 300 and 75 MHz.

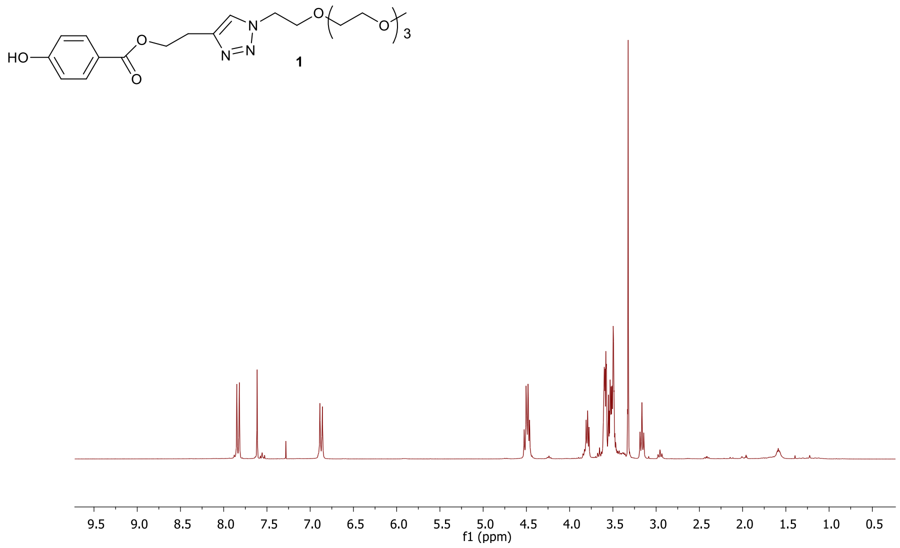

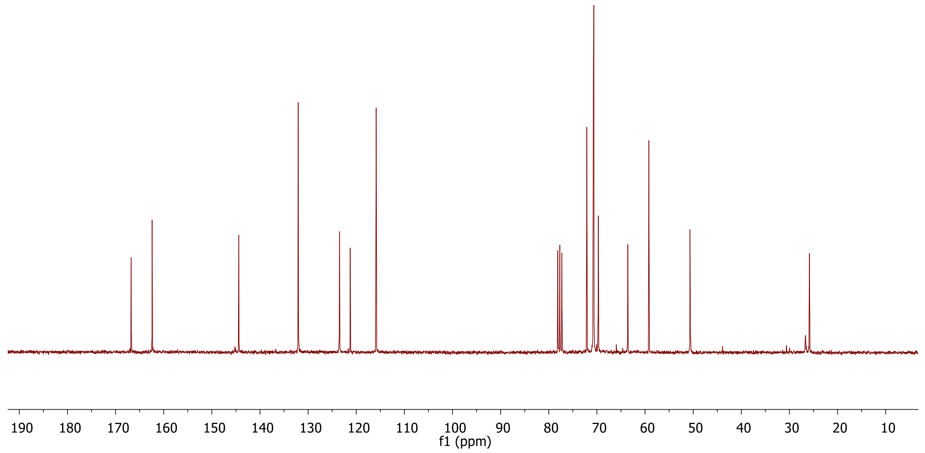

Figure S2. ^{1}H NMR and ^{13}C NMR spectrum of compound **2** in $CDCl_3$ at 300 and 75 MHz.

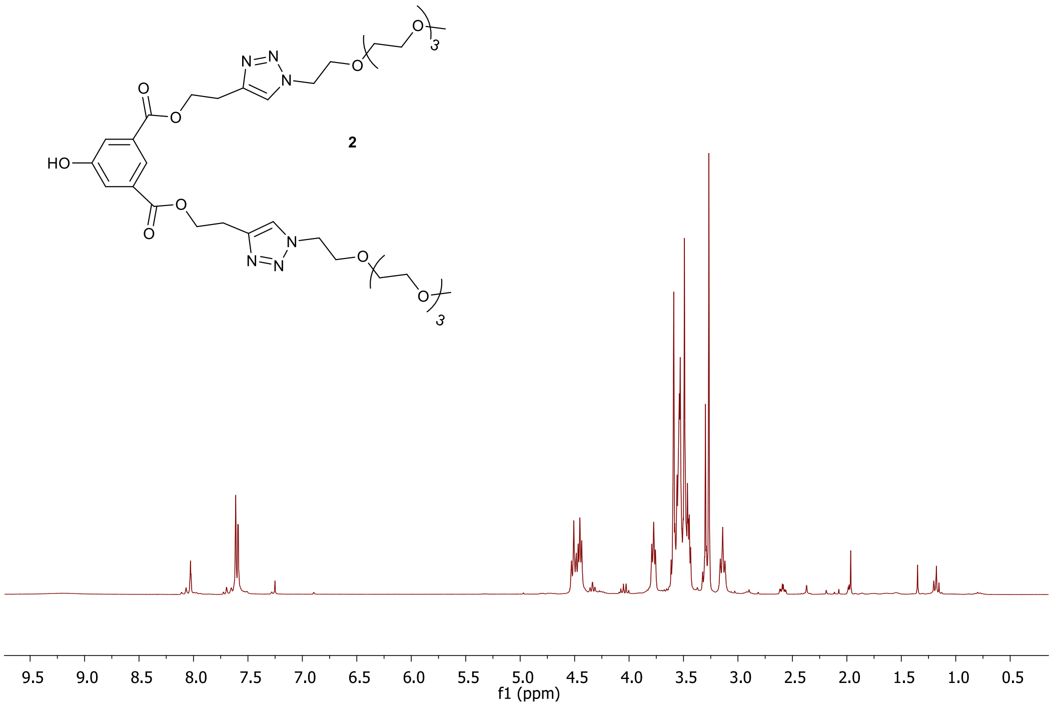

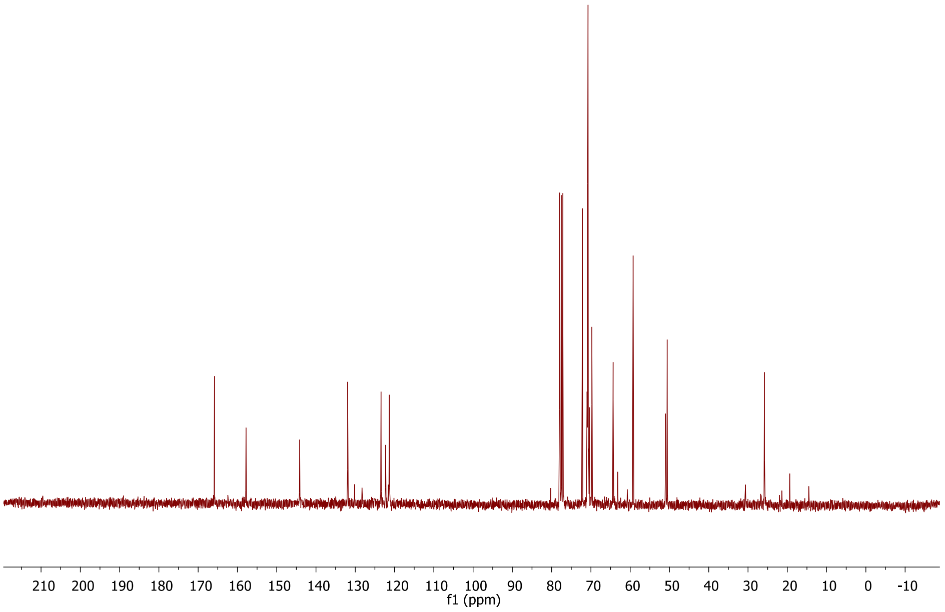

Figure S3. ^{1}H NMR and ^{13}C NMR spectra of 2,5,8,11-tetraoxatridecan-13-yl-4-methylbenzenesulfonate in $CDCl_3$ at 300 and 75 MHz.

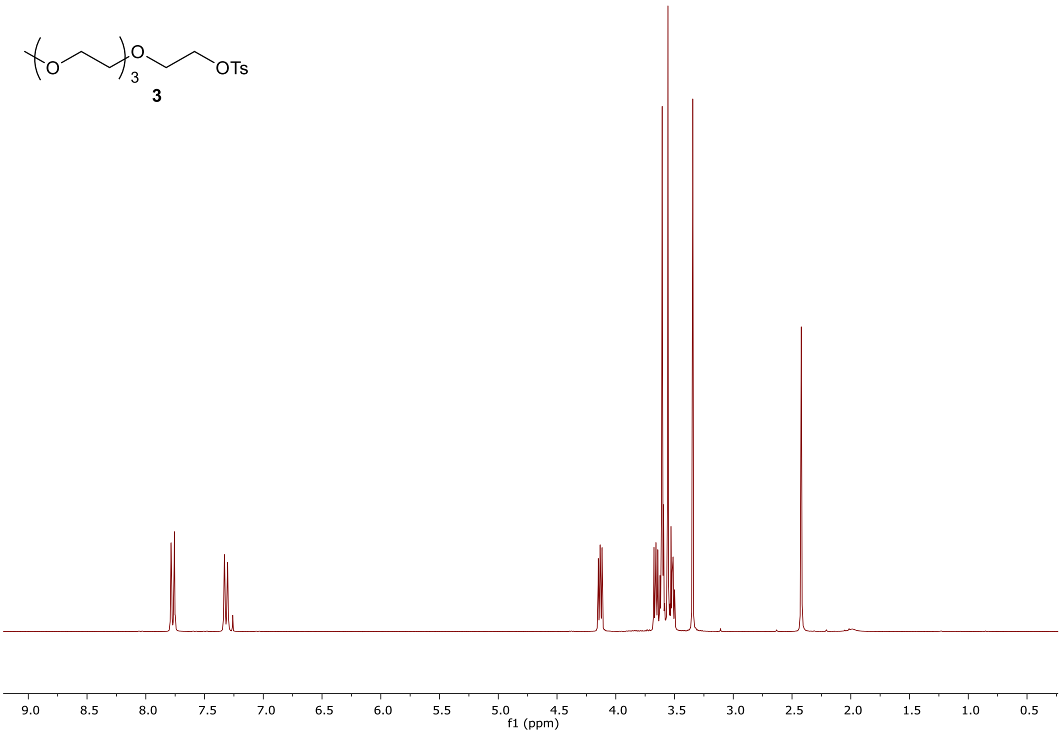

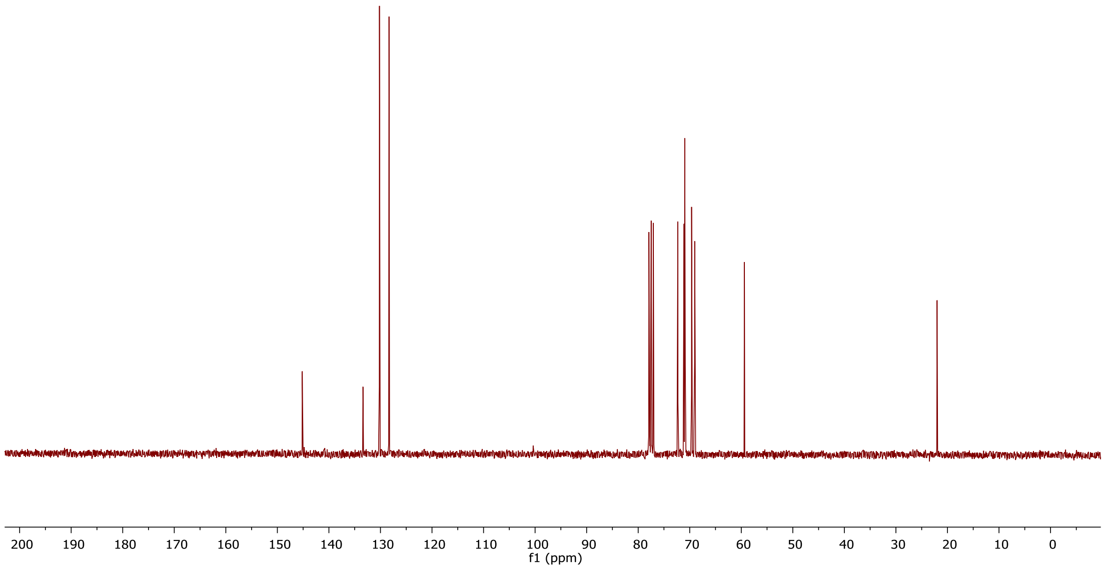

Figure S4. ^{1}H NMR and ^{13}C NMR spectrum of compound 13-azido-2,5,8,11-tetraoxatridecane (**3)** in $CDCl_3$ at 300 and 75 MHz.

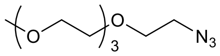

3

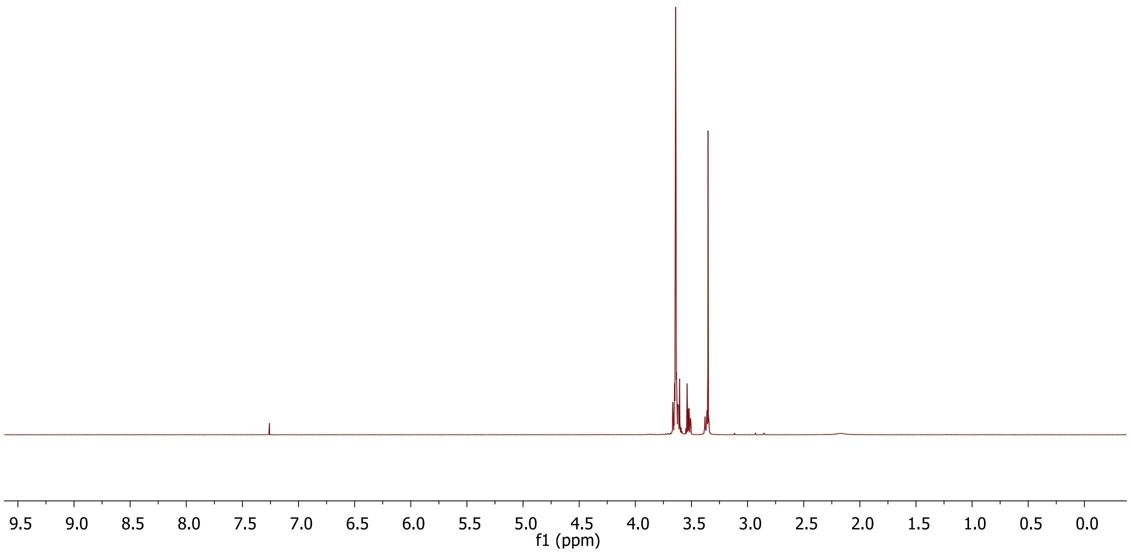

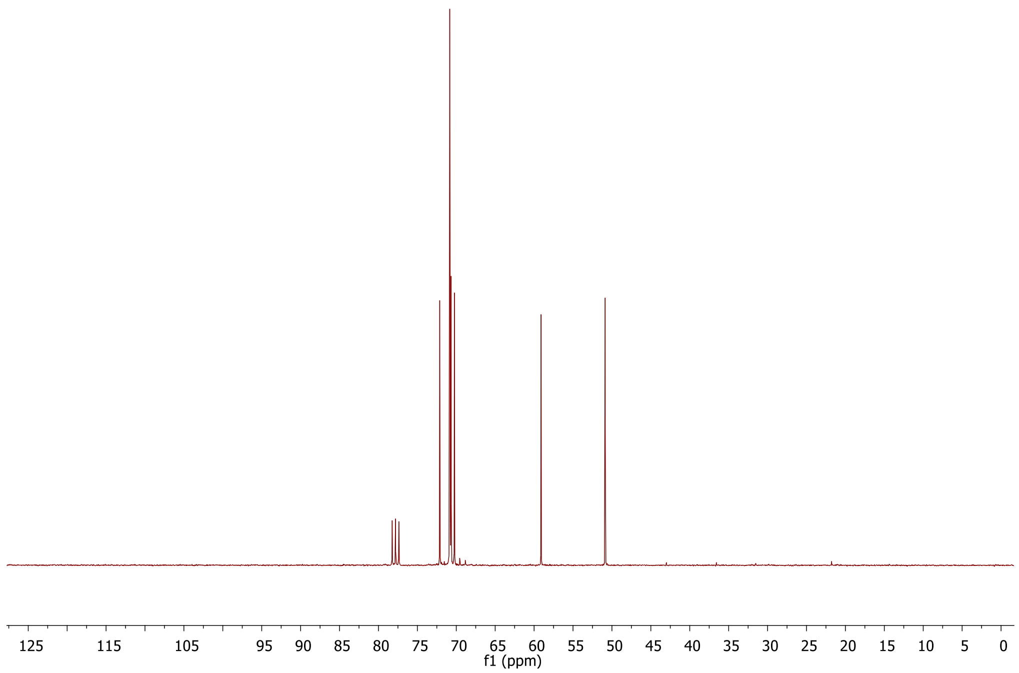

Figure S5. ^{1}H NMR and ^{13}C NMR spectrum of compound but-3-yn-1-yl 4-hydroxybenzoate (**4**) in $CDCl_3$ at 300 and 75 MHz.

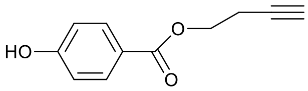

4

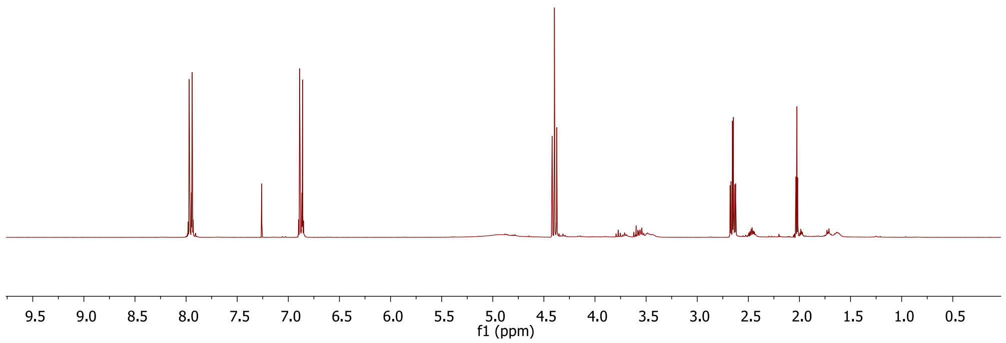

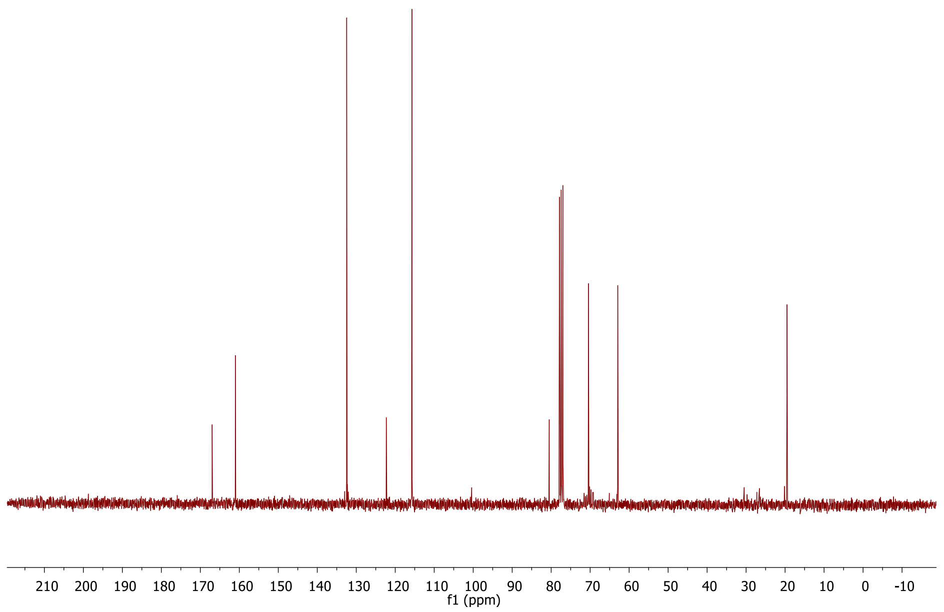

Figure S6. ^{1}H NMR and ^{13}C NMR spectrum of compound di(but-3-yn-1-yl) 5-hydroxyisophthalate (**5**) in $CDCl_3$ at 300 and 75 MHz.

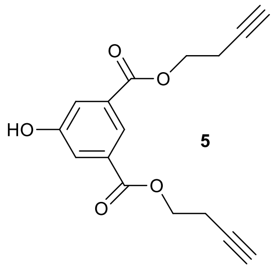

5

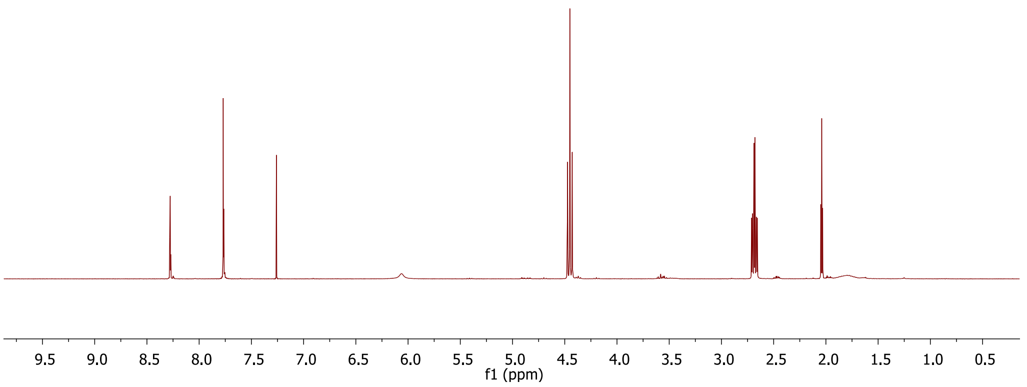

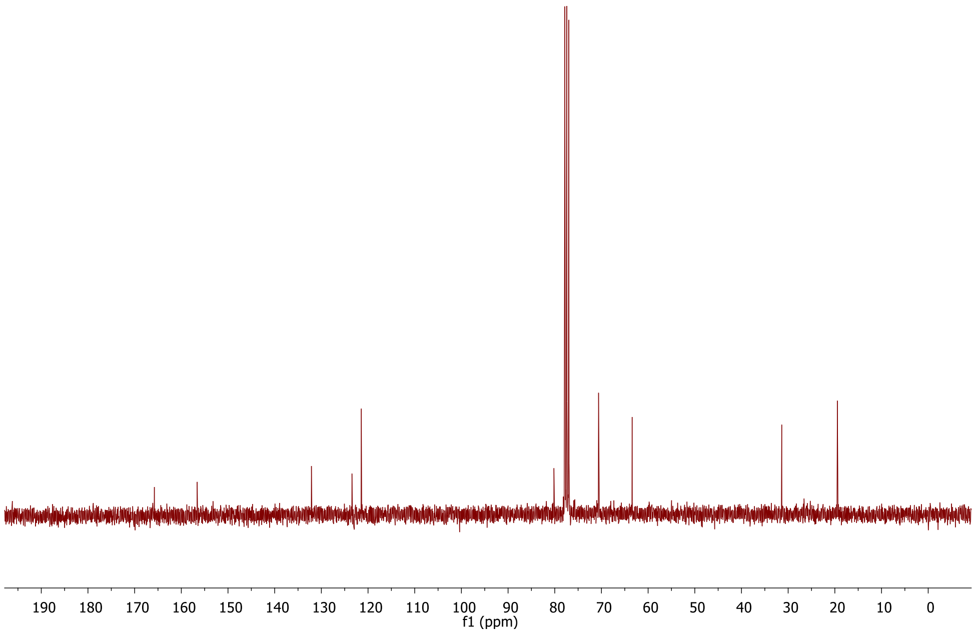

Figure S7. ^{1}H NMR and ^{13}C NMR spectrum of 4-(hydroxymethyl)phenyl benzoate (**6**) in $CDCl_3$ at 300 MHz and 75 MHz.

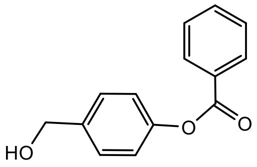

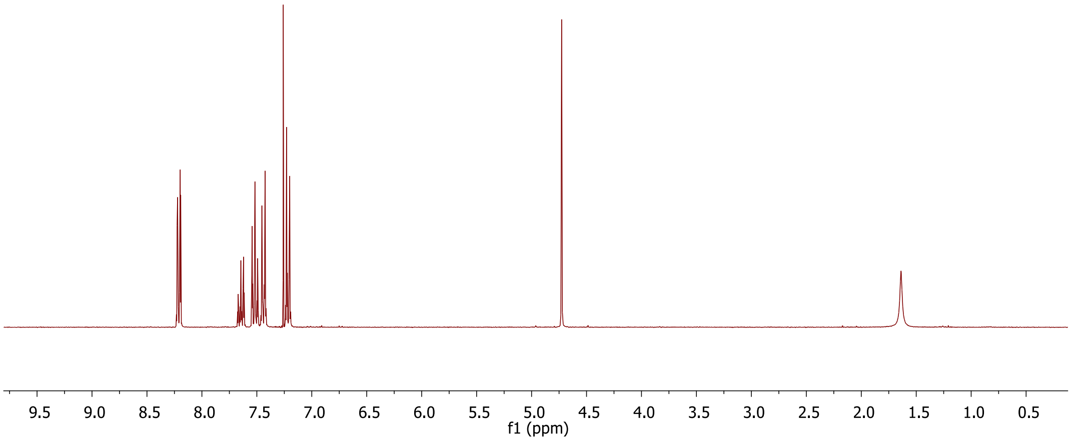

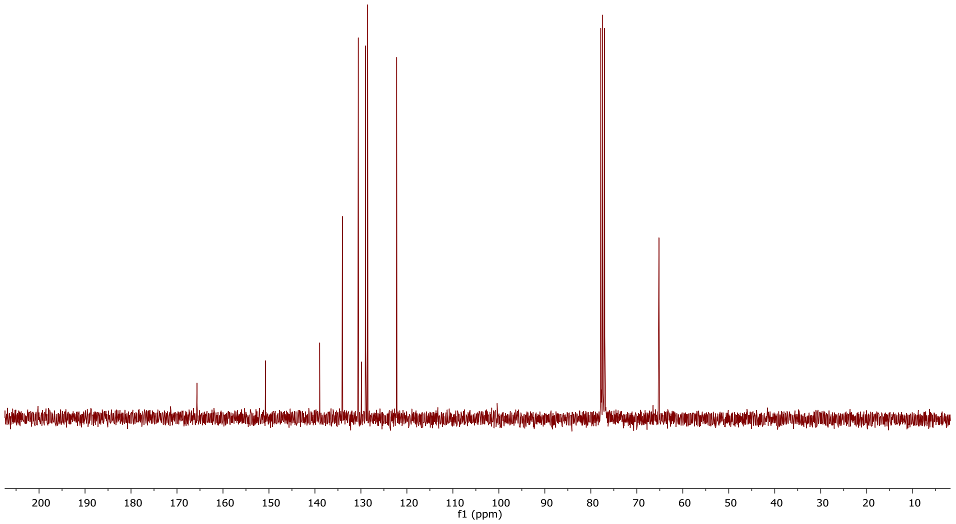

Figure S8. ^{1}H NMR and ^{13}C NMR spectrum 4-(hydroxymethyl)phenyl 1-naphthoate (**7**) in DMSO-d_6 at 300 and 75 MHz

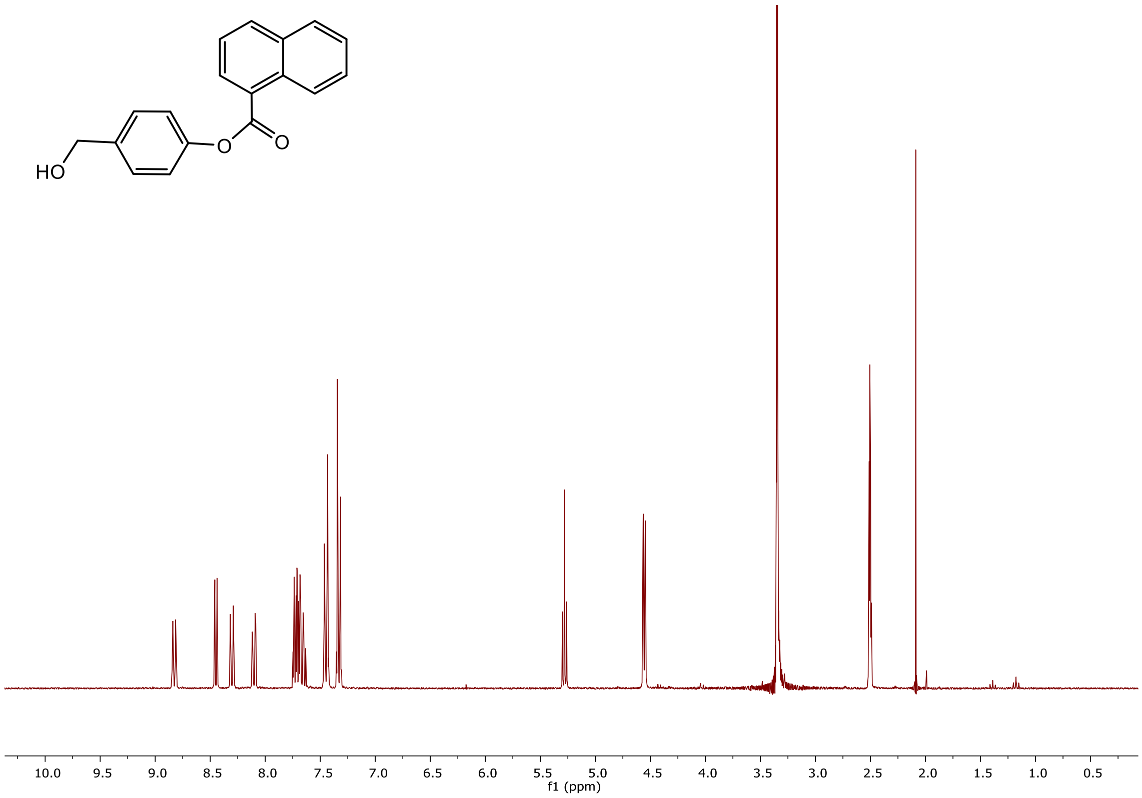

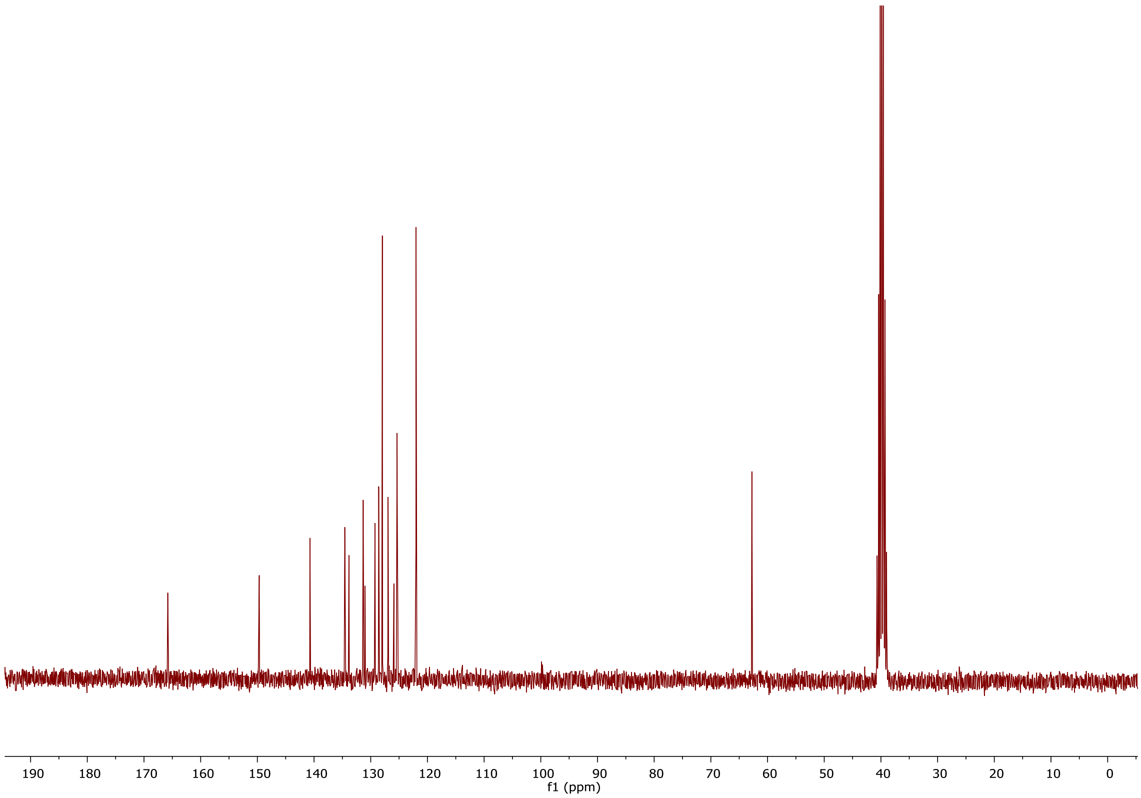

Figure S9. N_2 adsorption-desorption isotherms. When two isotherms have been combined, the curves have been y-shifted for clarity. Adsorption branch (■). Desorption branch (●).

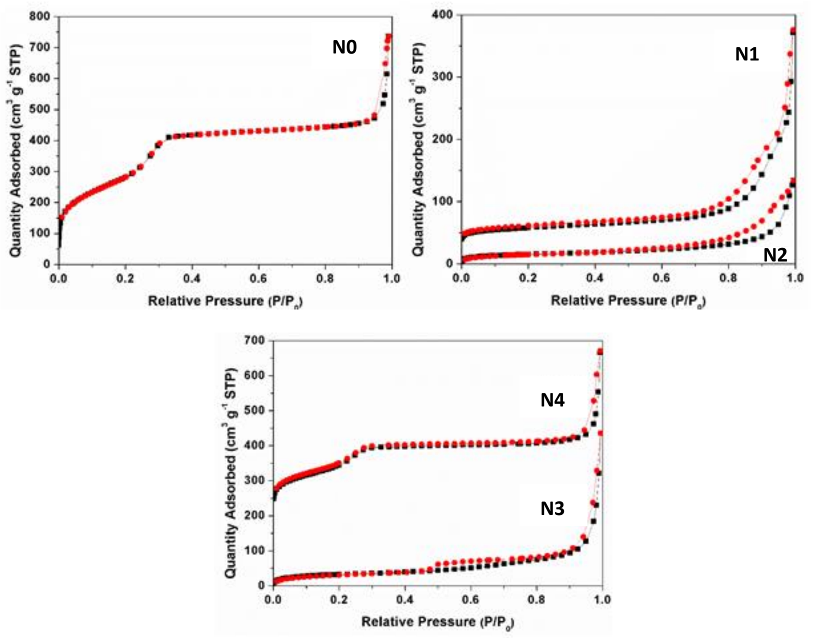

Figure S10. Molecular modeling of the molecular gates used in **N1**, **N2**, **N3** and **N4** preparation

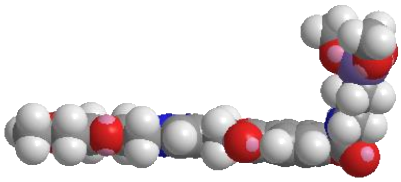

1

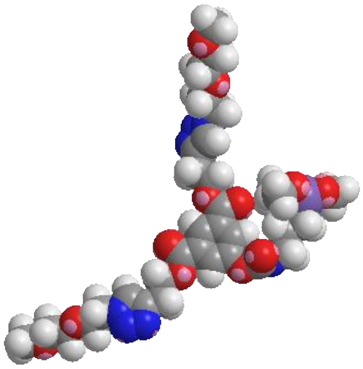

2

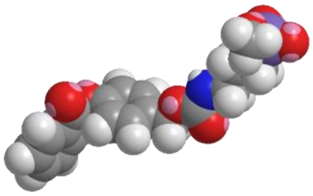

3

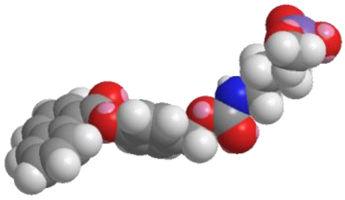

4

Figure S11. UV-vis spectrum of compound **3a** in EtOH/H_2O (50/50) at different concentrations.

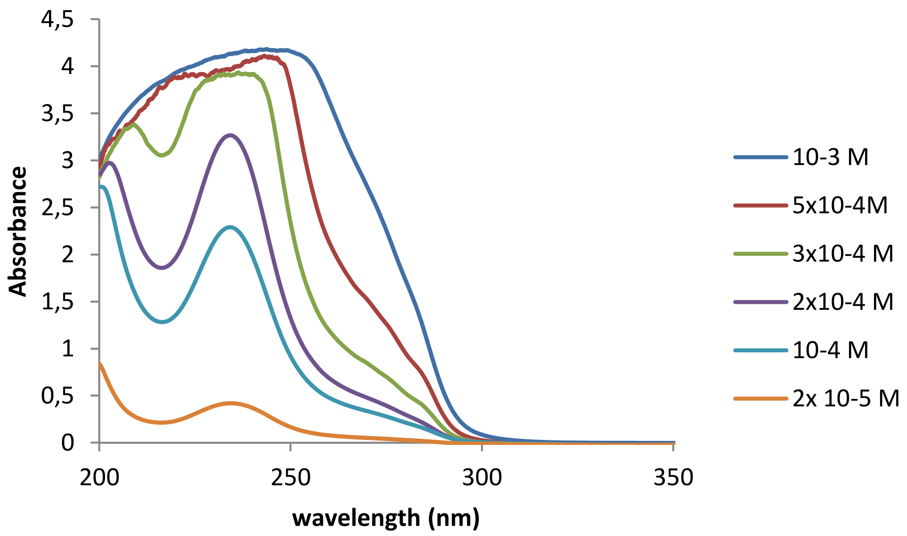

Figure S12. UV-vis spectrum of compound **4a** in EtOH/H_2O (50/50) at different concentrations.

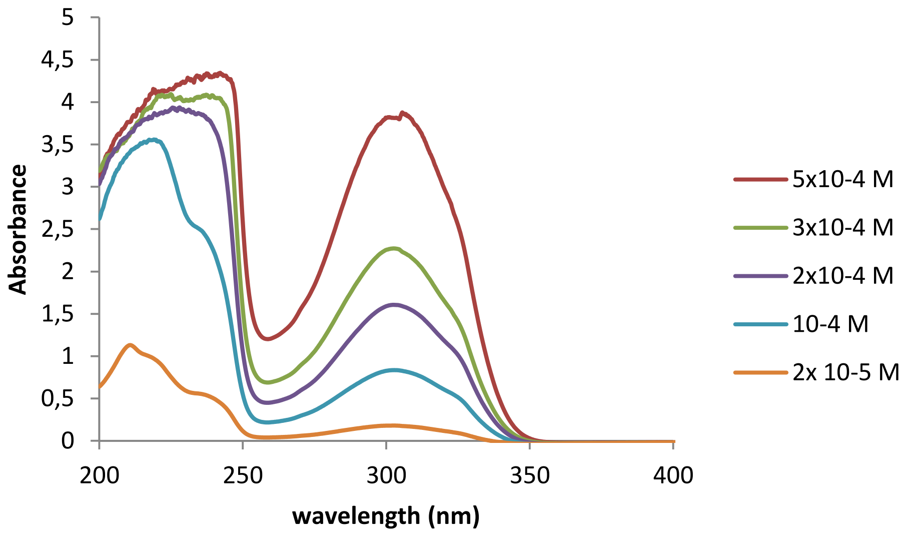

Figure S13. UV in solid phase of **N3**, **3a** and Rhodamine 6G

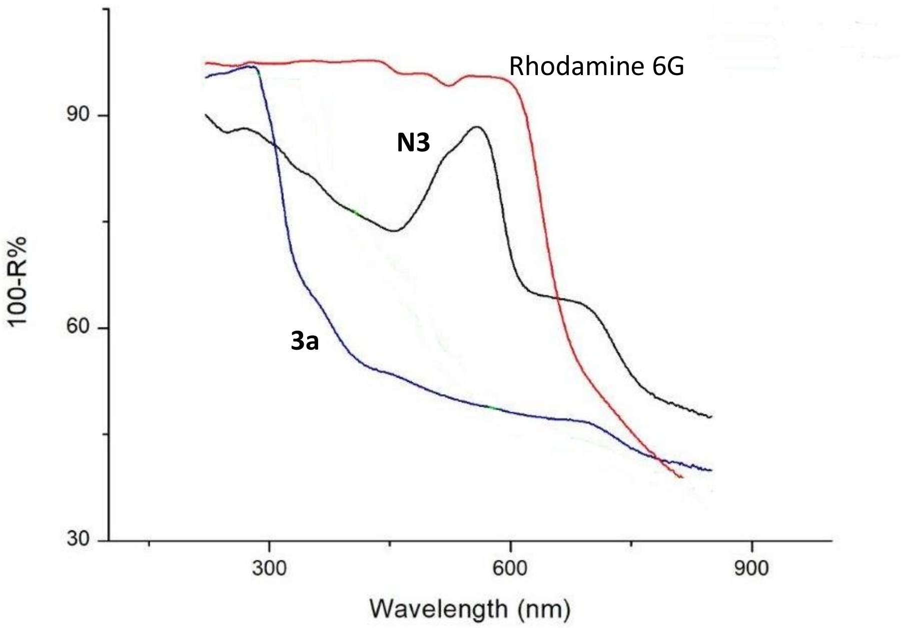

Supplement: Supplemental Material [file TSTA_A_1627173_SM9851.pdf]
